# Merlin’s disappearing act: *NF2* loss conjures pancreatic cancer survival in the hostile tumor microenvironment

**DOI:** 10.1172/JCI200909

**Published:** 2026-01-02

**Authors:** Sofia Ferreira, Laura D. Attardi

**Affiliations:** 1 Division of Radiation and Cancer Biology, Department of Radiation Oncology, Stanford University School of Medicine, Stanford, California, USA.; 2 Department of Genetics, Stanford University School of Medicine, Stanford, California, USA.

## Abstract

Pancreatic cancer cells “live on the edge,” starved of nutrients, compressed by abundant stiff stroma, and deprived of oxygen. In this issue, Xu et al. leveraged human pancreas organoid–based CRISPR screens to identify new driver genes in pancreatic ductal adenocarcinoma (PDAC) development. Neurofibromatosis type 2 (*NF2*) emerged as the top tumor suppressor, whose loss enhances PDAC malignancy. Inactivation of *NF2*, which encodes the protein Merlin, promoted growth factor independence and enhanced macropinocytosis upon nutrient deprivation. Thus, *NF2* status dictates the adaptability of pancreatic tumors under nutrient limitation, with *NF2* inactivation endowing PDACs with the ability to survive the constraints of the harsh tumor microenvironment.

Pancreatic ductal adenocarcinoma (PDAC) is the third leading cause of cancer deaths in the United States, with a 5-year survival rate of merely 13% (1, 2). PDAC is so deadly because it is often diagnosed at advanced stages, is largely unresponsive to existing therapies, and is associated with an aggressive, tumor-promoting microenvironment (3). Specifically, hypoxia, nutrient scarcity, mechanical compression, and dense stroma conspire against cell survival, selecting for cells with mutations that confer enhanced fitness in this harsh microenvironment (3). PDAC is driven by activating mutations in the *KRAS* oncogene, along with inactivating mutations in the *TP53*, *CDKN2A*, and *SMAD4* tumor suppressor genes (4, 5). While these tumor suppressors are frequently mutated in PDAC, there are numerous, less common genetic alterations observed in PDAC that might contribute to tumor development (4, 5). Uncovering these underappreciated mutations is crucial both for understanding PDAC biology and for identifying new therapeutic vulnerabilities in this devastating disease.

PDAC can arise either from acinar cells, the cells of the pancreas that secrete digestive enzymes, or ductal cells, the cells that line the conduits that carry digestive enzymes (6, 7). Notably, gene expression profiling has revealed that acinar cell–derived and ductal cell–derived PDACs in mice resemble the classical and basal-like subtypes of human PDAC (8). In this issue, Xu et al. used an organoid model they developed to identify new players in acinar cell–derived PDAC (9). In this model, termed KPT, they overexpressed *KRAS^G12V^* and inactivated both the *CDKN2A/P16* and *TP53* tumor suppressors in normal human pancreatic acinar cells and performed a pooled CRISPR-KO screen targeting 199 recurrently inactivated genes identified in patients with PDAC.

## *NF2* inactivation unleashes an aggressive PDAC phenotype

By performing CRISPR screens in an in vivo xenograft model, Xu et al. identified neurofibromatosis type 2 (*NF2*) as the top hit, with sgRNAs targeting *NF2* highly enriched in all samples (9). *NF2* encodes Merlin, a FERM domain protein that negatively regulates cell proliferation by serving as a linker between the plasma membrane and the actin cytoskeleton and modulating cell signaling (10). *NF2* has been primarily appreciated as a tumor suppressor through its mutation in the human familial cancer syndrome neurofibromatosis type II, which is characterized by schwannomas and meningiomas, although it is also commonly mutated in certain sporadic cancers such as mesotheliomas (11). Consistent with its importance in PDAC, Xu et al. showed using The Cancer Genome Atlas (TCGA) data that low *NF2* expression levels are associated with a poorer prognosis for patients with the classical subtype of PDAC.

**Figure 1:**
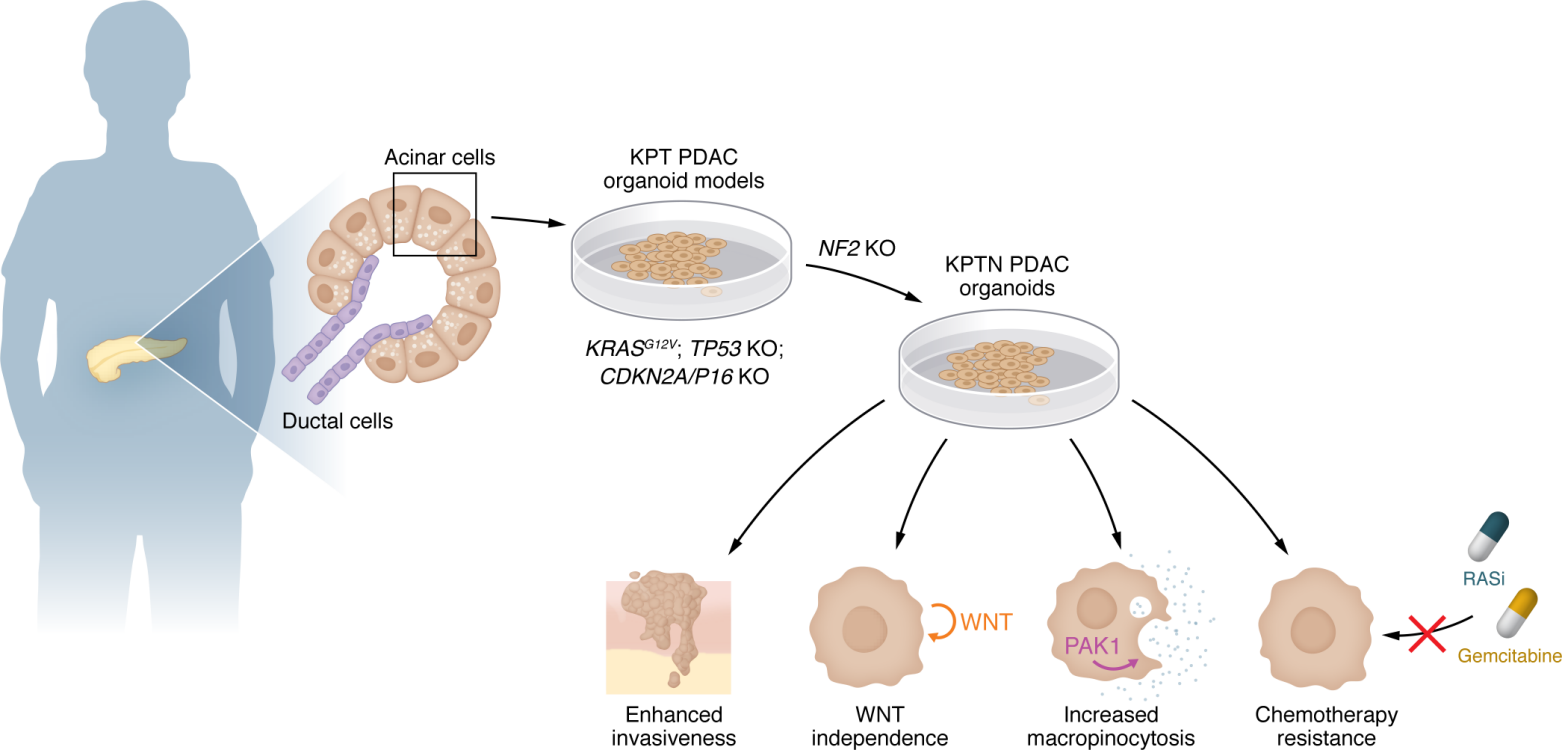
The role of *NF2* inactivation in PDAC. Using healthy human pancreatic acinar cells, Xu et al. (9) developed an organoid-based PDAC model by overexpressing *KRAS^G12V^* and inactivating the tumor suppressors *TP53* and *CDKN2A/P16* (termed the KPT model). A pooled CRISPR-KO screen identified *NF2* as a top tumor suppressor candidate. Inactivation of *NF2* in the KPT model (termed KPTN) led to multiple phenotypes associated with increased tumor aggressiveness and a poor prognosis, including enhanced invasiveness, WNT signaling independence, increased macropinocytosis via PAK1, and therapy resistance to a pan-RAS inhibitor (RASi) and gemcitabine.

Once the CRISPR screens had suggested the importance of *NF2* as a PDAC suppressor, Xu et al. further characterized the properties of KPT human acinar organoids with *NF2* KO (termed KPTN) (Figure 1). The KPTN organoids formed tumors more readily than did KPT organoids and prevailed in a coculture competition assay with KPT organoids. Moreover, KPTN tumors were larger, more invasive, and less differentiated than *KPT* tumors. They also were characterized by yes-associated protein (YAP) activation, consistent with previous work showing that *NF2* deficiency unleashes YAP/TAZ (transcriptional coactivator with PDZ-binding motif) signaling, providing an explanation for the enhanced aggressive phenotypes with *NF2* inactivation (10).

## *NF2* deficiency triggers metabolic rewiring, promoting tumor cell survival

Xu and colleagues’ gene expression profiling analysis revealed that not only YAP target genes but also WNT7B was induced with *NF2* deficiency (9). Early pancreatic precursor lesions and organoids are known to depend on exogenous WNT signaling for proliferation and maintenance (12). The upregulation of WNT7B allowed cells to bypass this dependency through autocrine WNT7B signaling, consistent with previous reports that Merlin restrains WNT/β-catenin signaling via FOXM1 in pancreatic cancer (13). KPTN organoids grew unperturbed even in low WNT conditions, whereas pharmacologic inhibition of WNT diminished their proliferative advantage. *NF2* inactivation thus reprogrammed acinar cell–derived organoids from WNT dependent to self-sufficient, enabling their growth even when cell-extrinsic cues disappeared.

Comparison of gene expression profiles driven by *NF2* inactivation in tumors in vivo and in organoids in vitro revealed the induction of programs associated with cellular starvation specifically in the context of tumors (9). These programs mirror the metabolic adaptations necessary for tumor progression in nutrient-poor conditions, as in vivo, and suggest cooperativity between the underlying genetics of the tumors and microenvironmental stressors. On the basis of these observations, the authors hypothesized that *NF2* deficiency endows tumors with the capability to survive in conditions of nutrient scarcity. Indeed, they found that KPTN organoids were able to grow effectively in low-nutrient media, in contrast to KPT organoids. To better understand this enhanced survival, Xu et al. examined macropinocytosis, a process that allows uptake of bulk nutrients from the extracellular space, and which is known to be important especially in RAS-driven cancers (14). They found that, relative to KPT organoids, KPTN organoids displayed enhanced macropinocytosis upon nutrient starvation, in a manner dependent on p21-activated kinase (PAK1). Blocking macropinocytosis abolished the survival advantage of KPTN organoids in response to nutrient depletion.

Given the metabolic rewiring that KPTN PDAC cells undergo, the authors speculated that there may be an effect on responses to therapy. Interestingly, they found that *NF2*-deficient cells, especially after acclimation to nutrient stress, exhibited resistance to both gemcitabine and a pan-RAS inhibitor, therapeutic agents used in PDAC. They found further that this resistance was at least partly mediated by antiapoptotic signaling, suggesting that during their evolution, KPTN PDACs develop strategies to evade apoptosis.

## Conclusions and future directions

Cancer progression relies on circumventing constraints that oppose its growth and persistence. Xu et al. show that *NF2* inactivation did more than remove brakes on proliferation. Loss of *NF2* reprogrammed acinar cell–derived PDAC into self-reliant, scavenging, apoptosis-resistant tumors. In a tumor where nutrients and oxygen are highly limited, such as PDACs, even minor advantages in nutrient uptake or signal autonomy may be decisive for tumor progression.

From a translational angle, *NF2* status could inform the stratification of patients with PDAC based on the predicted therapy response. Targeting Merlin-regulated processes, such as WNT7B secretion or PAK1-mediated macropinocytosis, may selectively exploit vulnerabilities of *NF2*-deficient PDACs. Although *NF2* mutations may be uncommon in PDAC, transcriptional suppression or functional inactivation of *NF2* may occur. Surveying clinical samples for *NF2* pathway disruption will help clarify the clinical relevance of these mechanisms.

Xu and colleagues demonstrate how integrating organoid-based functional genomics with in vivo modeling can reveal important facets of PDAC development. Beyond the biological and therapeutic implications derived from this study on Merlin, it will also be interesting in the future to conduct similar CRISPR screens in other contexts. For example, it will be worthwhile to repeat the acinar cell–derived PDAC organoid screens in immunocompetent mice to explore how PDACs developing in the backdrop of different mutations are influenced by crosstalk with the immune system. It will also be valuable to perform ductal cell–derived PDAC organoid screens to uncover relevant drivers of PDAC in this context, for a deeper understanding of tumor evolution from different cells of origin. Continued investigation into the biological basis of PDAC progression will be essential to ultimately understand how to conquer this aggressive and deadly cancer.

## Funding support

This work is the result of NIH funding, in whole or in part, and is subject to the NIH Public Access Policy. Through acceptance of this federal funding, the NIH has been given a right to make the work publicly available in PubMed Central.

- National Cancer Institute (NCI), NIH R35 grant CA197591 and P01 grant CA244114 (to LDA).
- Stanford Cancer Institute Innovation Award SPO 351119 (to LDA and SF).

## Version 1 01/02/2026

Electronic publication
